# Application of Dermal Skin Substitutes for Hand and Finger Palmar Soft Tissue Loss

**DOI:** 10.1097/GOX.0000000000002551

**Published:** 2019-11-27

**Authors:** Dominique Lucas, Damien Di Rocco, Camillo T. Müller, Abdo R. Jurjus, Wassim Raffoul, Pietro G. di Summa, William Watfa

**Affiliations:** From the * Plastic and Reconstructive Surgery Department, Centre Hospitalier Universitaire Vaudois, Lausanne, Switzerland; † Department of Anatomy, Cell Biology and Physiology, American University of Beirut, Beirut, Lebanon; ‡ Plastic, Reconstructive and Aesthetic Surgery Division, Saint George Hospital University Medical Center, Beirut, Lebanon.

## Abstract

Restoring function after traumatic defects of the palm is a reconstructive challenge, considering the need for flexible, elastic, and resistant skin. Dermal skin substitutes are biologically engineered materials composed of collagen and glycosaminoglycan, devoid of cellular structures. These biodegradable materials act as artificial dermis and stimulate neovascularization: they have been used for many years, mainly on the dorsal side of the hand and fingers, whereas the palmar side of the hand has been generally addressed by local flaps. In this study, we described our experience with dermal skin substitutes in two cases of palmar defects associated with exposed tendinous structures. Coverage of palmar defects in hand and fingers with dermal substitute and split thickness skin graft was performed on two patients. Both patients presented palmar-only loss of tissue (traumatic palmar amputation in the first patient and degloving-type injury in the second patient). Range of motion, functional outcomes, and satisfaction and aesthetical results were evaluated. The resulting skin showed good quality, thickness, pliability, and Disabilities of the Arm, Shoulder, and Hand (DASH) score. Additionally, the patients regained full range of motion and reported high satisfaction. The association of split thickness skin graft with dermal substitutes in palmar traumatic hand showed optimal functional and aesthetic outcomes. Although being more adapted to dorsal loss of substance, collagen-based dermal substitutes can also be useful reconstructive tools in palmar defects with exposed structures and could be used to a larger extent in the future.

## INTRODUCTION

Palmar skin differs from the dorsal skin by its thickness, rich innervations, and its inferior extensibility. Skin grafting is widely considered as a simple, affordable, and effective way of correcting skin defects resulting from trauma. However, the major limitation of skin grafts appears when facing defects that include tendons, bone, or even nonbiological tissues.

Despite the numerous flap possibilities (rotation, advancement, transposition), disadvantage of flaps includes the potential morbidity of the donor site such as unsightly scars and stiffness (cross finger, inguinal, and thenar flaps) and less extensibility. Free flaps require a longer operating time and the need of a microsurgical team. In addition, the paddles of these different flaps remain quite different from the palmar skin of the hand from an aesthetic and architectural level.

Dermal substitutes have been used for approximately 15 years in burn management and reconstructive surgery,^1^ with increasing popularity. The rationale is to provide a support that mimics the missing dermis, allowing better split thickness skin graft (STSG) take.^2^

Whether or not dermal substitutes are suitable for palmar defect reconstruction is the aim of this work, which also reports long-term functional and aesthetic outcomes.

### Case 1

**Fig. 1.**
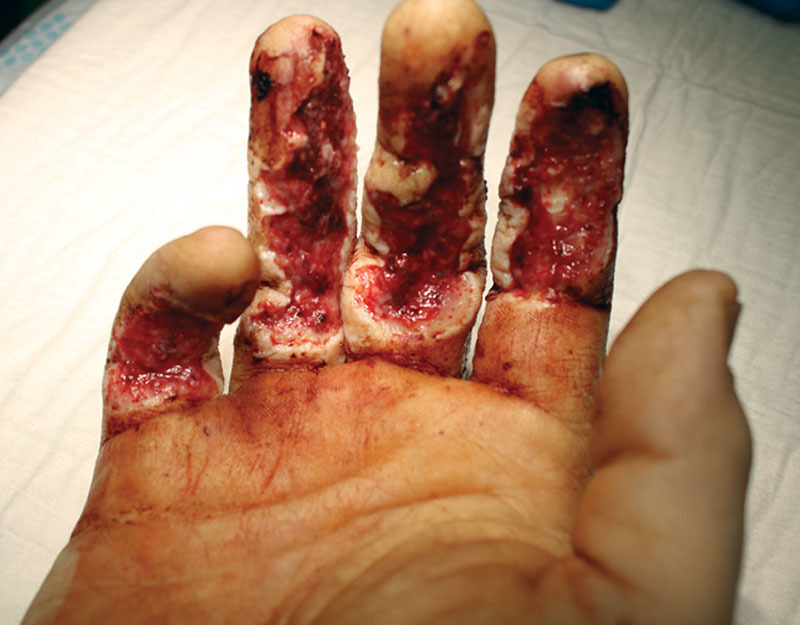
Palmar defect of the right hand in patient 1 before surgical debridement.

Patient 1, a 38-year-old left-handed, manual worker, nonsmoker, was a victim of an occupational accident while cutting wood, resulting in loss of substance of the palmar face of the index, middle, ring, and little fingers of his right hand (Fig. 1). Physical examination showed exposed flexor tendons of index, middle, ring, and little fingers. The flexor digitorum profundus (FDP) of the ring finger was ruptured in zone I. Good vascularization of fingertip was present. Radiological findings showed an intraarticular multifragmented fracture of the distal interphalangeal (DIP) of the fourth finger.

Surgical exploration showed a 2-cm defect of the ulnar digital nerve of the index, and a 3-cm defect of both middle finger ulnar/radial digital nerves in addition to several flexor tendons areas (1–3 mm) devoid of paratendon. In zone I of the middle finger, the DIP and distal phalanx were exposed devoid of periosteum.

**Fig. 2.**
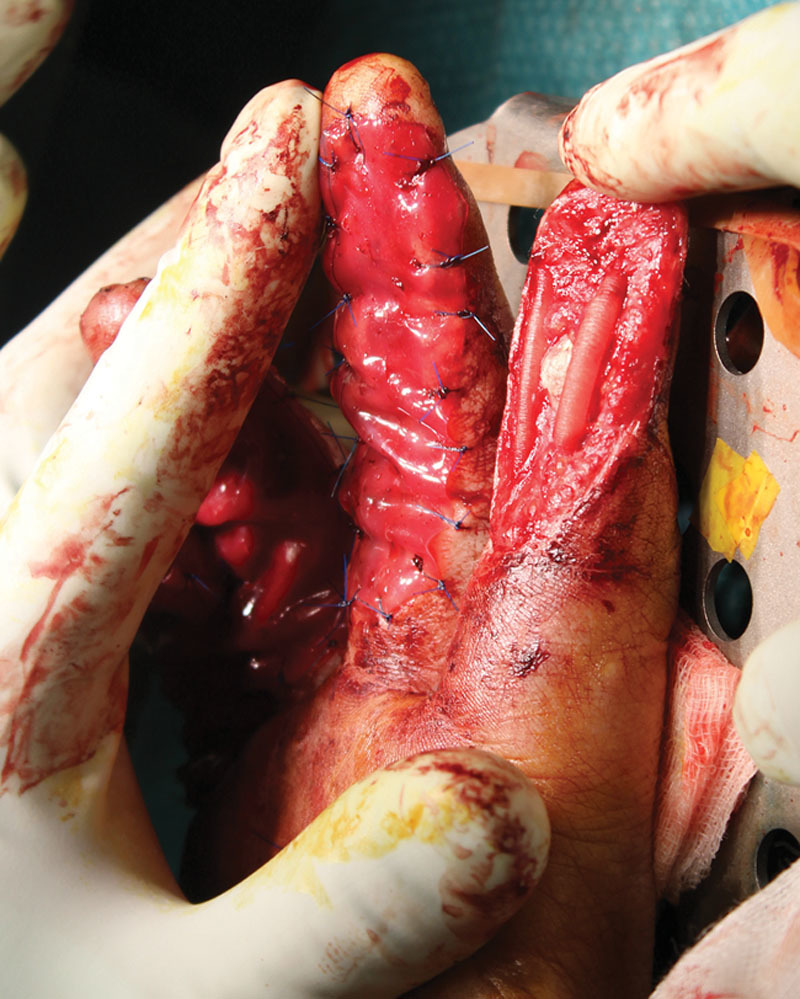
Application of neurotubes in patient 1.

**Fig. 3.**
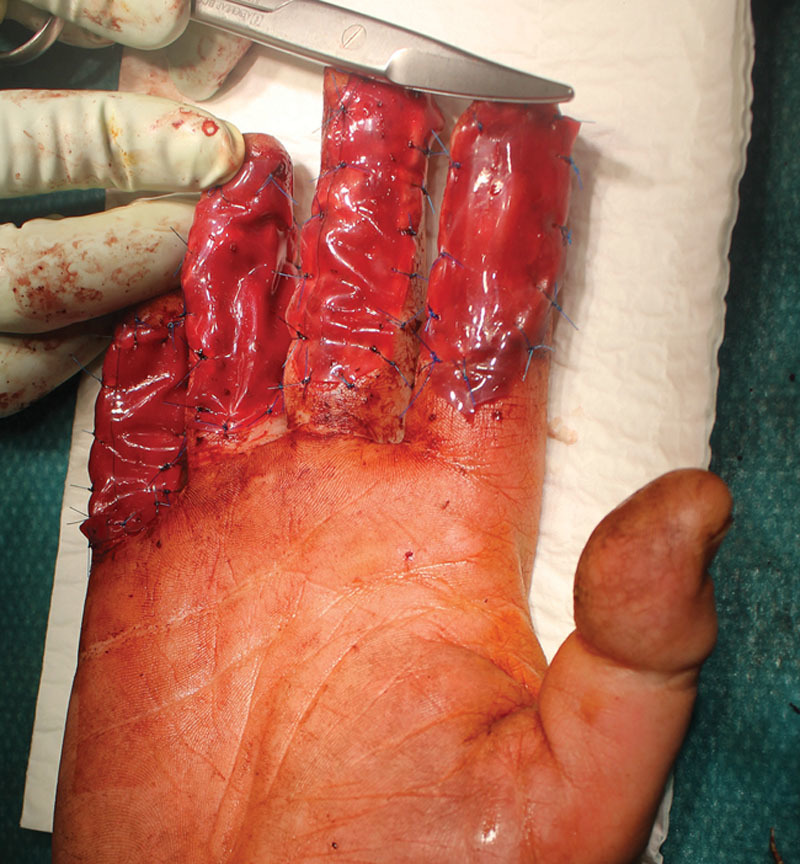
Application of integra over neurotubes in patient 1.

On the same day, after debridement and washout, DIP joint arthrodesis of the fourth finger by the Kirschner wiring and repair of the fourth FDP were performed. Neurotubes were placed on the radial and ulnar digital nerves of the index and on the ulnar digital nerve of the fourth finger. A double-layer Integra (Integra LifeSciences Services, Plainsboro, NJ ) was applied on the Neurotubes (NeuraGen) (Figs. 2 and 3). The patient was discharged at day 2 postoperative with immediate occupational therapy twice a week (active and passive work) and weekly dressing change. After 1 month, we removed the superficial layer of the Integra and covered with STSG (harvested from the thigh) over the palmar face of the 4 affected digits.

**Fig. 4.**
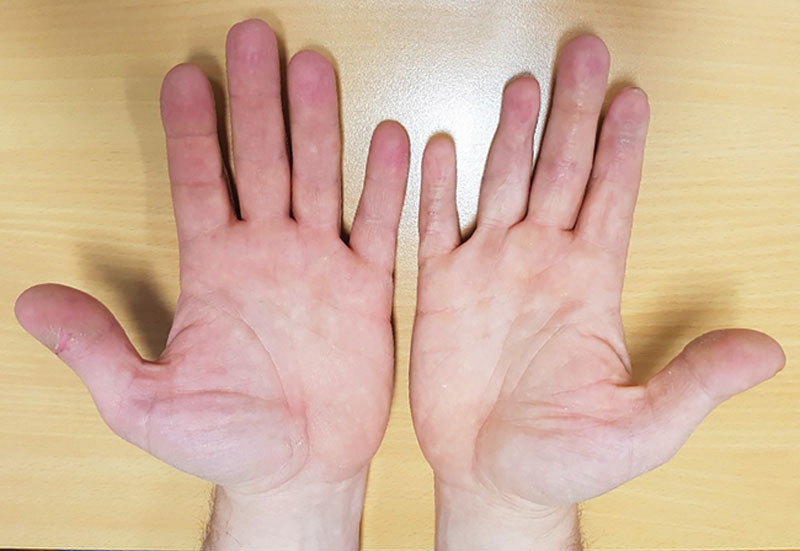
Cosmetic appearance at 1 year in patient 1.

At 8-month follow-up, the patient had resumed work. Physical examination showed a Weber 2PD of 4 mm (radial ring finger), 3 mm (ulnar ring finger), and 3 mm (ulnar index). Jamar dynamometer was of 39 kg for the right hand (versus 55 kg for the left hand). The tip-fold distance was of 1 cm for the index, middle, and little fingers, and 12 mm for the ring finger (DIP arthrodesis). DASH score was 6. The patient reported high satisfaction with regard to cosmetic result. Skin quality and texture were similar to the contralateral hand with good aesthetic outcome (Fig. 4).

### Case 2

Patient 2, a 51-year-old, right-handed, manual worker, nonsmoker, was a victim of an occupational accident while sanding wood, resulting in a circumferential avulsion of the left hand, involving distal thirds of the second, third, fourth, and fifth metacarpals and phalanges.

Surgical exploration on the day of admission showed multilevel interruption of the palmar neurovascular pedicles on all fingers at the metacarpophalangeal (MCP) level, except for the middle finger where both pedicles were visualized up to mid-P2. Many large areas (up to 1 cm^2^), especially at the extensor tendon system, lacked paratenon. After extensive debridement and abundant washout, Epigard (Biovision, Germany) was applied as temporary dressing.

At day 10, after obtaining a good granulation tissue, a sheath of Matriderm 1 mm (MatriDerm, MedSkin Solution Dr. Suwelack AG, Billerbeck, Germany) was applied on the defect area with an STSG. After 48 hours, occupational therapy twice a week was started (active and passive work) and weekly dressing change was performed.

However, at 3-month follow-up, we had to perform trans-DIP amputation on the index, middle, and fifth fingers and trans-DIP on the fourth finger with distal end coverage by thin skin graft.

At 8 months posttrauma, examination showed 0-20-60° mobility for MCP of D2, D4, and D5, 0-30-60° for MCP of D3 and 0-35-60° for the proximal interphalangeal (PIP) of D4. The skin appearance was good, and the patient had a functional thumb–index grasp with the ability to perform fine gestures (close buttons on his shirt, move small objects). He felt pressure on the palmar surface of the fingers with his eyes closed. DASH score was 14.

## DISCUSSION

Recently, the use of skin grafting in combination with dermal matrices (DM) has gained some popularity. The benefits of DM in the coverage of loss of substance even when structures are exposed are widely described.^3–6^ In this aspect, ACell (ACell, Inc., Columbia, Maryland) one of the xenograft biological dermal substitutes (derived from porcine urothelium) has successfully been used to cover nerve or tendons.

In a recent publication, we showed that the combination of STSG with MatriDerm substantially decreased postoperative morbidity at the donor site after raising radial forearm free flaps.^7^ Matriderm associated to STSG guaranteed a significantly better functional, sensory and cosmetic result, when compared with full thickness skin graft. DMs were able to limit adhesions between different planes while allowing early mobilization due to myofibroblasts migrating to the wound bed decreases, thus preventing secondary retraction.^8,9^ From our experience in burns surgery, we prefer using double layer integra when facing traumatic loss of substance to give time to inflammation and bacterial contamination to decrease and therefore provide a graftable wound bed. The combination of Neurotube and Integra may seem ambitious, but we have always had good results in the past. Care was taken to meticulously prepare both proximal and distal stumps and avoiding leaving more than a 10-mm gap in the tube.^10^

Ellis and Kulber^3^ reported a very high satisfaction in their series with no complications. There were no major postoperative complications, except for late osteomyelitis of the third phalanx amputated in the second patient, with a good response to antibiotic therapy. We also acknowledge that in this same case, the use of DM instead of a groin flap was debated and long discussed with the patient. After a positive Laser Doppler for sufficient vascularization in the distal phalanges and the patient’s preference for a shorter and less laborious reconstruction, we finally opted for Matriderm use.

Both patients were able to benefit from early mobilization with occupational therapy, without the need for strict postoperative immobilization, which helped in preventing joint stiffness. The amplitudes of movement and range of motion at distant follow-up were satisfactory.

## CONCLUSIONS

DMs have already found their place in palmar burns of the hand, but very few traumatic cases have been described in the literature. The ability to apply DMs to “nongraftable” structures allows breaking some reconstructive rules such us grafting on tendon devoid of paratendon, on bone without periosteum and on biomaterials such as Neurotube. According to these experiences, even if limited in time and number, these adjunct tools could be considered for potential use as a second or third line procedure when flaps or grafts are not possible or contraindicated in palmar hand defects.
